# Evaluation of psychometric properties of patient-reported outcome measures frequently used in narcolepsy randomized controlled trials: a systematic review

**DOI:** 10.1093/sleep/zsac156

**Published:** 2022-07-07

**Authors:** Aaron Schokman, Yu Sun Bin, Diana Naehrig, Janet M Y Cheung, Kristina Kairaitis, Nick Glozier

**Affiliations:** Faculty of Medicine and Health, University of Sydney, Camperdown, NSW, Australia; Sleep Theme at Charles Perkins Centre, University of Sydney, Camperdown, NSW, Australia; Sleep Theme at Charles Perkins Centre, University of Sydney, Camperdown, NSW, Australia; Faculty of Medicine and Health, University of Sydney, Camperdown, NSW, Australia; Faculty of Medicine and Health, University of Sydney, Camperdown, NSW, Australia; Department of Respiratory and Sleep Medicine, University of Sydney at Westmead Hospital, Westmead, NSW, Australia; Ludwig Engel Centre for Respiratory Research, Westmead Institute for Medical Research, Westmead, NSW, Australia; Faculty of Medicine and Health, University of Sydney, Camperdown, NSW, Australia

**Keywords:** narcolepsy, cataplexy, psychometric properties, patient-reported outcome measures, randomized controlled trials, cosmin, systematic review, reliability, responsiveness, validity

## Abstract

**Study Objectives:**

To systematically determine subjective and objective outcome measures used to measure the efficacy of narcolepsy interventions in randomized controlled trials (RCTs) in adults and children and assess psychometric properties of patient-reported outcome measures (PROMs) used.

**Methods:**

We searched bibliographical databases and clinical trial registries for narcolepsy RCTs and extracted objective and subjective outcome measures. If PROMs were used, we searched for psychometric studies conducted in a narcolepsy population using bibliographical databases and appraised using Consensus-based Standards for the Selection of Health Measurement Instruments (COSMIN) guidelines.

**Results:**

In total, 80 different outcome measures were used across 100 RCTs. Epworth Sleepiness Scale (ESS) (*n* = 49) and Maintenance of Wakefulness Test (*n* = 47) were the most frequently used outcome measures. We found 19 validation studies of 10 PROMs in narcolepsy populations. There was limited evidence for validity or responsiveness of the ESS; yet sufficient reliability (pooled ICC: 0.81–0.87). Narcolepsy Severity Scale (NSS) had sufficient reliability (pooled ICC: 0.71–0.92) and both adult and pediatric versions had sufficient discriminant validity (treated/untreated). Content validity was only evaluated in pediatric populations for ESS-CHAD and NSS-P and rated inconclusive. Quality of evidence of the psychometric studies for all scales ranged from very low to low.

**Conclusions:**

Although recognized by regulatory bodies and widely used as primary outcome measures in trials, there is surprisingly little evidence for the validity, reliability, and responsiveness of PROMs frequently used to assess treatment efficacy in narcolepsy. The field needs to establish patient-centered minimal clinically important differences for the PROMs used in these trials.

**Statement of Significance**

This is the first systematic review that explores both the outcome measures used in randomized controlled trials (RCTs) of people with narcolepsy and the psychometric properties of frequently used patient-reported outcome measures (PROMs). Narcolepsy interventions focus exclusively on treating symptoms; thus, knowing what outcome measures are used in efficacy studies is important if patients’ expectation of treatment is to be met. Patient-reported outcome measures are frequently used in narcolepsy RCTs; however, there is limited evidence showing their appropriateness for use (i.e. validity, reliability, responsiveness). Furthermore, psychometric studies on existing PROMs or the development of ones that are narcolepsy-specific are needed before we can be confident that interventions are efficacious.

## Introduction

Five symptoms characterize narcolepsy: excessive daytime sleepiness (EDS), cataplexy, hypnogogic/hypnopompic hallucinations, sleep paralysis, and disrupted nocturnal sleep [1]. The presence of cataplexy (sudden loss of skeletal muscle tone triggered by a strong emotion such as laughter) differentiates between the two subtypes of narcolepsy: narcolepsy with cataplexy—narcolepsy type 1 (N1); and narcolepsy without cataplexy—narcolepsy type 2 (N2) [2]. The most common approach to treating narcolepsy is pharmacological intervention, with current medications focused entirely on treating symptoms [3]. Nevertheless, those with narcolepsy continue to experience negative impacts on quality of life and daily function from symptoms, despite receiving standard treatment [3, 4].

Randomized controlled trials (RCTs) are the gold standard for establishing treatment efficacy [5]. Choosing outcome measures that accurately capture symptoms of narcolepsy is important not only to interpret the effects of treatment correctly but also to ensure the results are valuable to clinicians, people with narcolepsy, and other decision makers [6]. Outcome measures are generally categorized as either objective or subjective. Objective measures are quantifiable and independent of an individual’s opinion or experience (e.g. Maintenance of Wakefulness Test [MWT]), whereas subjective measures are based on personal experience (e.g. Epworth Sleepiness Scale [ESS]). An important subset of subjective measures are patient-reported outcome measures (PROMs). These are typically short, easy-to-answer questionnaires completed by patients and are designed to capture the patient experience of specific concepts/constructs such as symptoms and the impact of a health condition in a way that is considered meaningful to patients.

The ESS has been used as the primary endpoint for EDS in efficacy trials and is considered sufficient evidence for regulatory approval of narcolepsy treatments [7–9]. PROMs are often created to measure complex and often unobservable constructs based on individual perspectives. Care must be taken to ensure a PROM actually measures the construct of interest, particularly if used in another population or for a different purpose than the one it was designed for [10]. The FDA has published guidelines on PROM use in therapeutic development, requiring evidence of the validity of PROMs to support medical product labelling claims [11]. Documented characteristics of the PROM are required (e.g. the number of items, and the population for intended use), including evidence showing its adequacy in terms of measurement properties, commonly referred to as psychometric properties (e.g. content validity, internal consistency). A PROMs usefulness can be determined by assessing its validity (i.e. the construct the PROM purports to measure is truly what is being measured), reliability (i.e. the PROM is free from measurement error), and responsiveness (i.e. the PROM is able to detect meaningful change) [12]. The *Co*nsensus-based *S*tandards for the selection of health *M*easurement *In*struments (COSMIN) guidelines provide uniform terminology of psychometric properties and standards/criteria by which psychometric properties of a PROM can be assessed [10].

The importance of showing adequate content validity of a PROM is stressed by the FDA, EMA, and COSMIN over other psychometric properties [11, 12]. Content validity is “the degree to which the content of an instrument is an adequate reflection of the construct to be measured” [10]. Using a PROM in another population than the one it was designed for and validated in requires evidence that the two populations’ perception of the construct being measured is the same. Individual questions that make up a PROM need to be relevant to the specific construct that is being measured (specific to each population and context of use) and comprehensive enough that the PROM thoroughly reflects a respondent’s perception of the construct [10]. Conversely, insufficient content validity can affect how other psychometric properties are interpreted [10]. For example, while a high Cronbach’s α demonstrates high internal consistency, it does not guarantee that the construct of interest is accurately captured or that all-important concepts are included. Similarly, high test–retest reliability or high responsiveness does not guarantee construct validity [10].

Our aim was to evaluate the extent to which PROMs are used in RCTs to measure treatment success in a narcolepsy population and the adequacy of the PROMs used in a two-staged systematic review:

Stage 1: To identify the objective and subjective outcome measures used to measure narcolepsy treatment in RCTs involving adults and children.
Stage 2: To evaluate the published evidence of psychometric properties of PROMs frequently used in narcolepsy RCTs.

## Methods

This two-stage systematic review was prospectively registered with the PROSPERO International Prospective Register of Systematic Reviews (CRD42020209827) and followed the Preferred Reporting Items for Systematic Reviews and Meta-Analyses (PRISMA) 2020 guidelines and checklist [13]. This review also utilized the COSMIN initiatives guidelines for conducting a systematic review of PROMs in a target population [10, 14]. This includes guidance on searching for studies of each measurement property of PROMs and criteria by which the methodological quality of each study and the results are assessed.

### Stage 1: To identify the objective and subjective outcome measures used to measure narcolepsy treatment in RCTs involving adults and children

#### Eligibility criteria

Publications and clinical trial protocols describing RCTs investigating the efficacy of treatment intervention in people with narcolepsy were eligible for review. Participants of eligible studies were either adults or children diagnosed with narcolepsy (either type 1 or 2) using either the International Classification of Sleep Disorders (ICSD) or the Diagnostic and Statistical Manual of Mental Disorders 5th edition (DSM-5). No criteria were placed on the type of intervention used in RCTs, nor was any restriction placed on the date of publication. Publications or protocols written in a language other than English were excluded. If a publication cited a clinical trial protocol, the publication was excluded in favor of the clinical trial protocol.

#### Information sources and search strategy

Medline (Ovid), Embase (Ovid), PsycINFO (Ovid), CINAHL, and Scopus and clinical trial registries (www.clinicaltrials.gov, www.clinicaltrialsregister.eu, and www.anzctr.org.au) were searched on the 24th of May 2022. The search strategy for published RCTs combined a Cochrane filter used to identify RCTs (sensitivity-maximizing version) and keywords/MeSH terms specific to narcolepsy [15]. Clinical trial records were searched for intervention studies that involved narcolepsy or cataplexy-specific populations. Our search strategy can be found in Supplementary A.

#### Study selection

Title, abstract, and full-text screening of eligible articles were independently performed by two reviewers (A.S. and D.N.) using Covidence, an online systematic review tool [16]. Disagreements were discussed among reviewers, and consensus was reached, with a third reviewer (N.G.) adjudicating. Studies with both a Clinicaltrials.gov record and published articles were only included once by comparing clinicaltrials.gov identifiers. Multiple publications from a single RCT were limited to the primary paper describing the trial results and main outcome measures used.

#### Data items

Outcome measures that were used to measure treatment efficacy were extracted from eligible studies and categorized as primary or secondary outcome measures independently by two authors (A.S. and D.N.) using information contained in study records. In the event published journal articles did not explicitly identify a measure as primary or secondary, the paper’s content and aims were reviewed (A.S., D.N., and N.G.) until a consensus was reached. Coprimary outcome measures were each counted as a primary outcome measure.

#### Classification of outcome measures

Outcome measures identified were classified as either objective or self-reported measurements (authors A.S. and N.G.). Self-reported measures were further classified as either (1) PROMs if it assessed the status of a patient’s health condition using a standardized bank of items and responses were made directly by the patient, without interpretation by another person, or proxy report (except if the patient was a child) or (2) used another method such as a visual analog scale, diary, or answered by another person (i.e. physician completing the Clinical Global Impressions Scales) [11].

### Stage 2: To evaluate the published evidence of psychometric properties of PROMs frequently used in narcolepsy RCTs

#### Eligibility criteria

##### patient-reported outcome measures

PROMs identified in stage 1 of this review that either assessed narcolepsy symptoms and/or associated disability and function were eligible for inclusion in stage 2. PROMs were included if used as an outcome measure in (1) at least two narcolepsy RCTs or (2) at least one narcolepsy RCT and were developed specifically for use in a narcolepsy population. Instances where a PROM may have been used (e.g. sleep diary) but no explicit PROM mentioned (e.g. consensus sleep diary) were not eligible. PROMs assessing constructs not specific to narcolepsy symptoms or associated disability (e.g. quality-of-life, function, mental health, etc.) were also excluded. Characteristics of identified PROMs were extracted from original development studies and presented using the recommended COSMIN tabular format (Table 1).

**Table 1.** Characteristics of the patient-reported outcome measures that are used in atleast two RCTs investigating treatment efficacy in people with narcolepsy or used in atleast one narcolepsy RCT and developed specifically for narcolepsy

PROM (reference to first article)	Construct(s)	Target population	Recall period	(Sub)scale(s) (number of items)	Response options	Range of scores	Original language
Stanford Sleepiness Scale (SSS)	Situational sleepiness, sleepiness at a given time	Any adult	At time of measure	1 (1)	(1) Feeling active and vital, (2) Functioning at a high level, (3) Relaxed; awake, (4) A little foggy, (5) Fogginess, (6) Sleepiness, (7) Almost in Reverie	1–7 (response option is score)	English
Epworth Sleepiness Scale (ESS) [18]	Average sleep propensity in daily life	Adults with EDS or suspected EDS	Prior month	1 (8)	(0) Would never doze, (1) Slight chance of dozing, (2) Moderate chance of dosing, (3) High chance of dosing	0–24; (higher scores indicate higher likelihood the scorer will fall asleep during the day)	English
Epworth Sleepiness Scale—Children and adolescent (ESS-CHAD) [19]	Average sleep propensity in daily life	Children and adolescents with EDS or suspected EDS			(0) Would never fall asleep, (1) Slight chance of falling asleep, (2) Moderate chance of falling asleep, (3) High chance of falling asleep		
Karolinska Sleepiness Scale (KSS) [20]	Situational sleepiness, sleepiness at a given time	Any adult	At time of measure	1 (1)	(1) Extremely alert, (2) Very alert, (3) Alert, (4) Rather alert, (5) Neither alert nor sleepy, (6) Some signs of sleepiness, (7) Sleepy—but no effort remaining awake, (8) Sleepy, but some effort to stay awake (9) Very sleepy, great effort to stay awake, (10) Extremely sleepy, can’t keep awake	1–10 (score represents sleepiness at given time)	English
Pittsburgh Sleep Quality Index (PSQI) [21]	Sleep quality, habits, and disturbances	Any adult	Prior month	7 (19)	(0) Very good, (1) Fairly good, (2) Fairly bad, (3) Very bad	0–21 (subscales scored 0–3). Higher scores indicates worse sleep quality	English
Brief Fatigue Inventory (BFI) [22]	Severity and impact of cancer-related fatigue	Patients with fatigue due to cancer and cancer treatment	Prior 24 h	1 (9)	11-Point numeric scale, with higher scores indicating higher levels of fatigue and interference with daily life	0–10 (global fatigue score obtained by averaging items. Higher scores indicate more fatigue)	English
Pediatric Daytime Sleepiness Scale (PDSS) [23]	Daytime sleepiness	Students aged 5–17 years old	No time frame	1 (8)	(0) Never, (1) Seldom, (2) Sometimes, (3) Frequently, (4) Always	Higher scores indicate increased sleepiness and are associated with poorer educational outcomes	English
Narcolepsy Symptom Assessment Questionnaire (NSAQ) [24]	Changes in narcolepsy status and symptoms	Individuals with narcolepsy*	Prior 24 h	26 questions across various domains	Varying (questions ask scorer to rate symptoms as increased, decreased, or remains the same, 5-point Likert scales)	—	English
Narcolepsy Severity Scale (NSS) [25]	The severity of main narcolepsy symptoms	Adults diagnosed with narcolepsy type 1	Prior month	1 (15)	Varying (4 and 6 Likert scale)	Mild (0–14), moderate (15–28), severe (29–42), and very severe (43–57)	French
Narcolepsy Severity Scale—Paediatric (NSS-P) [26]	The severity of main narcolepsy symptoms	Children diagnosed with narcolepsy type 1		1 (14)		Mild (0–14), moderate (15–28), severe (29–42), and very severe (43–54)	

PROM: Patient Reported Outcome Measure, ESS: Epworth Sleepiness Scale, ESS-CHAD: Epworth Sleepiness Scale—Children and Adolescent, PSQI: Pittsburgh Sleep Quality Index, NSS: Narcolepsy Severity Scale, NSS-P: Narcolepsy Severity Scale-Pediatric, SSS: Stanford Sleepiness Scale.

##### types of studies

Psychometric studies of eligible PROMs were required to have been published in a peer-reviewed journal, with the full-text available in English. Cross-cultural adaptation studies were also included. Studies that investigated the psychometric properties of a PROM in the context of diagnosing narcolepsy (i.e. discriminative validity) were excluded.

##### participants

To be eligible, psychometric studies had to be conducted in a population diagnosed with narcolepsy (N1 or N2) using ICSD-1–3 or DSM criteria. Studies conducted in a mixed population (i.e. participants with various sleep disorders) were included if an analysis of the psychometric properties using a narcolepsy subsample was described. Studies utilizing both adult and children/adolescent populations were included

#### Information sources and search strategy for validation studies of included PROMs

Published studies investigating content validity or other measurement properties of included PROMs were searched for on the 24th of May 2022. Studies were searched for using Medline (Ovid), Embase (Ovid), PsycINFO (Ovid), CINAHL, and Scopus bibliographical databases using an amalgamation of COSMIN recommended search strategies and Cochrane narcolepsy-specific search strategy (Supplementary A).

#### Data extraction and analysis of psychometric properties of PROMs using the COSMIN checklist

One reviewer (A.S.) screened all title/abstract and full-text articles to determine eligibility. The full-text evaluation of the screened articles and data extraction were conducted independently by two authors (A.S. and Y.S.B.) using the COSMIN checklist. The checklist consists of questions that assess content validity and eight other measurement properties: (1) structural validity, (2) internal consistency, (3) cross-cultural validity/measurement invariance, (4) reliability, (5) measurement error, (6) criterion validity, (7) hypotheses testing for construct validity (convergence and discriminative), and (8) responsiveness to change (in response to intervention) [10]. The COSMIN checklist was completed in three stages. The study design (methodology used) and potential risk of bias of each study exploring measurement properties of PROMs were rated using a four-point scale (excellent, good, fair, poor), with the lowest rating of any of the questions used as the overall rating.

Second, the results from each study of any one measurement property of a PROM are rated against the criteria for what is considered a “good measurement property” (Supplementary Table S1). The criteria assess both the framework used to assess the measurement property and the result obtained against a specific standard (e.g. was Cronbach’s α used to assess internal consistency AND was the result ≥0.70). A three-point rating scale is used for each result (sufficient, indeterminant, insufficient), with the ratings pooled together to give an overall score for the quality of the measurement property for each PROM (Supplementary Table S4).

Finally, an overall score of the quality of evidence for each pooled result of a measurement property is determined (Supplementary Table S3) using a modified version of the Grading of Recommendations Assessment, Development, and Evaluation (GRADE) [17]. A four-point scale is used (high, moderate, low, very low), with each study starting with a “high” rating. The rating combines the first two components of the COSMIN checklist, and each study is subsequently downgraded based on the potential risk of bias in the studies, inconsistencies in the pooling of results, imprecision (i.e. total sample size), and indirectness (i.e. used partly in other populations or settings) (Supplementary Table S2).

## Results

### Stage 1: To identify the objective and subjective outcome measures used to measure narcolepsy treatment in RCTs involving adults and children

**Figure 1.**
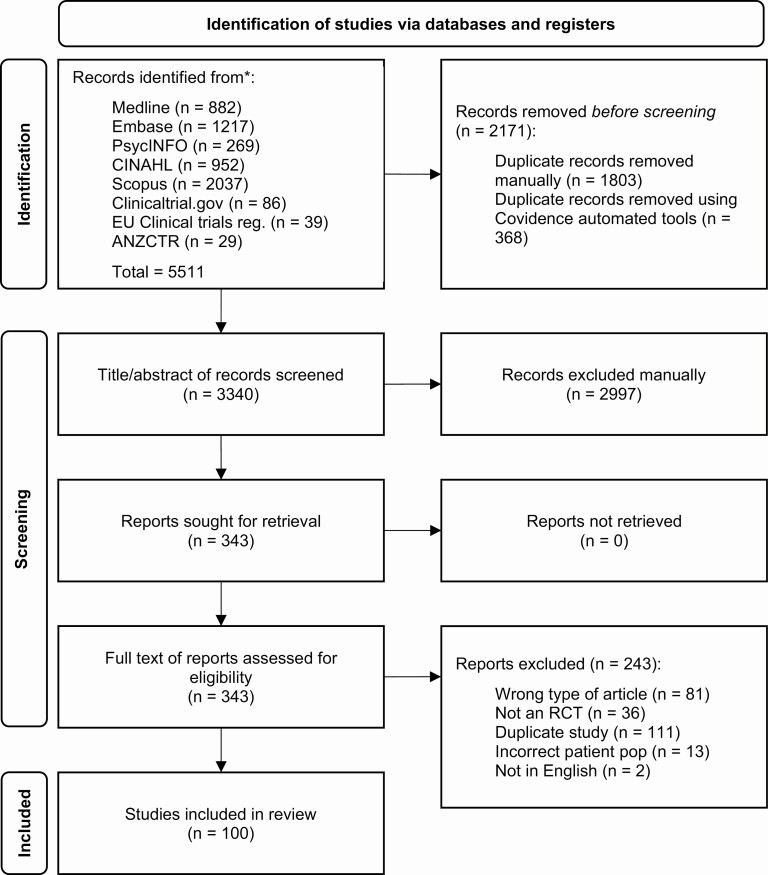
Preferred Reporting Items for Systematic Reviews and Meta-Analyses (PRISMA) diagram for identifying eligible randomized controlled trials in a narcolepsy population. RCT: Randomized controlled trial.

The systematic search identified 5511 records, of which 5357 were sourced from bibliography databases and 154 from clinical trial records (Figure 1). Following the removal of duplicates, 3340 records underwent title and abstract screening. A total of 343 records were selected for full-text screening, from which 100 RCTs conducted in a narcolepsy population were identified and included.

**Figure 2.**
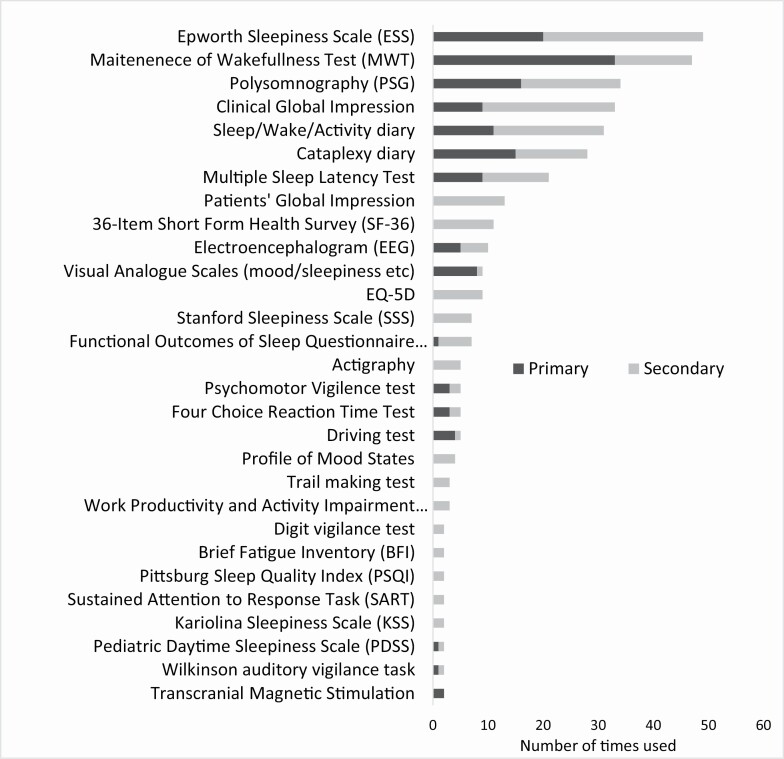
Outcome measures used in two or more RCTs in people with narcolepsy (as identified in the first stage of this systematic review) stratified by use as either a primary or secondary outcome measure.

Across these 100 RCTs, we identified 80 unique outcome measures used to assess treatment efficacy. Outcome measures used in at least two RCTs can be found in Figure 2, stratified by their use as a primary or secondary outcome measure. Thirty-eight (48%) of the measures used were objective, and 42 (52%) were subjective. A PROM, the ESS (*n* = 49), was the most frequently used of all outcome measures in these RCTs [18]. The most common objective outcome measures used were the MWT (*n* = 47, and also the most common primary outcome measure *n* = 33 studies), polysomnography (PSG) (*n* = 34) and multiple sleep latency test (MSLT) (*n* = 21), while the most common subjective measures were the ESS (*n* = 49), clinical global impressions scale (*n* = 33) and sleep/wake/activity diaries (*n* = 31) (Figure 2). Nonstandardized weekly diaries (where the patient or parent records the number and severity of cataplexy attacks) were the most used subjective outcome measure for the symptom of cataplexy (*n* = 28).

Of the 100 RCTs identified, four were conducted in a pediatric population (age < 18 years). A cataplexy diary was the most common primary outcome measure (*n* = 2) used, followed by the MSLT (*n* = 1), CGI-C (*n* = 1), and PDSS (*n* = 1). The ESS-CHAD was used once as a secondary outcome measure.

We identified 10 PROMs as having either been used in two or more RCTs or used in at least one RCT and developed to assess symptoms/associated disability of narcolepsy (Table 1). Of these, the ESS was the only PROM to be used in two or more RCTs, having been used a total of 20 times as a primary outcome measure to assess narcolepsy symptoms and/or associated disability. Only one other PROM was used as the primary outcome measure: the Pediatric Daytime Sleepiness Scale (PDSS) [27].

### Stage 2: to evaluate the published evidence of psychometric properties of PROMs frequently used in narcolepsy RCTs

**Figure 3.**
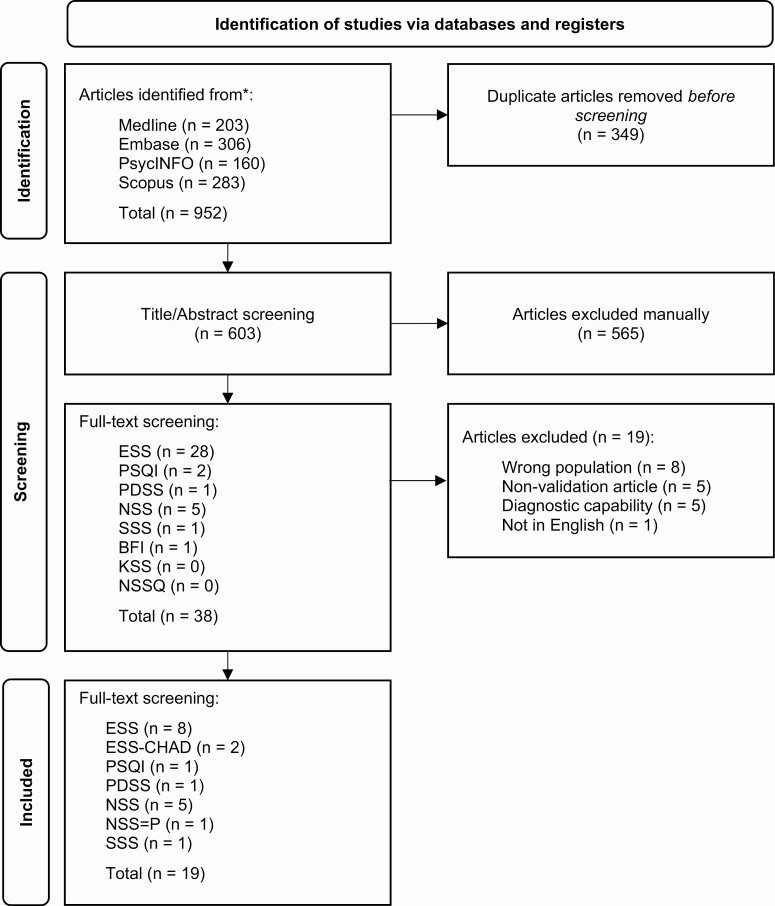
Preferred Reporting Items for Systematic Reviews and Meta-Analyses (PRISMA) diagram for identifying eligible psychometric studies of frequently used patient-reported outcome measures in narcolepsy randomized controlled trials. RCT: randomized controlled trial, ESS: Epworth Sleepiness Scale, ESS-CHAD: Epworth Sleepiness Scale—Children and Adolescent, PSQI: Pittsburgh Sleep Quality Index, NSS: Narcolepsy Severity Scale, NSS-P: Narcolepsy Severity Scale-Pediatric, SSS: Stanford Sleepiness Scale, BFI: Brief Fatigue Inventory, KSS: Karolinska Sleepiness Scale, NSSQ: Narcolepsy Sleep Status Questionnaire.

We systematically searched for psychometric validation studies of the 10 PROMs frequently used in RCTs and identified 952 records sourced from bibliography databases (Figure 3). Most of the articles found were related to the ESS (62%). Following the removal of duplicates, 603 records underwent title and abstract screening. A total of 38 records were selected for full-text screening. Nineteen validation studies of the 10 PROMs were found. Most studies (*n* = 9) related to the ESS, with six being retrospective analyses of RCT data and two being validation studies of a modified version of the ESS specific for children and adolescents (ESS-CHAD).

Characteristics of the ten PROMs frequently used in narcolepsy RCTs and included in stage 2 of this review can be found in Table 1. A summary of the 19 studies that explore the psychometric properties of these PROMs can be found in Table 2.

**Table 2.** Validation studies of commonly used patient-reported outcome measures in RCTs investigating treatment efficacy in people with narcolepsy

			Population with narcolepsy				Instrument administration		
PROM	Ref	Setting	*n*	Age, mean (SD, range), yr	Gender % female	Disease	Inclusion criteria	Country	Language
ESS	[35]	Retrospective analysis of clinical trial	231	36.2 (13.2, —)	65%	N1, N2	Diagnosis using ICSD-3 or DSM-5, required to have baseline mean sleep latency <25 min on the MWT and usual nightly total sleep time ≥6 h. Key exclusion criteria included usual bedtime later than 1:00 am, an occupation requiring nighttime or variable shift work, or any other clinically relevant medical, behavioral, or psychiatric disorder associated with EDS	United States	English
	[38]								
	[36]	Retrospective analysis of clinical trial	95	Intervention group A = 38.2 (14.1, —), Intervention group B = 39.3 (15.4, —)	45%	N1, N2	ICSD-2 and a baseline score of ≥14 on the ESS	Switzerland	—
	[39]	Retrospective analysis of two clinical trials	228	Trial 1: 38.6 (—) Trial 2: 40.5 (—) Range of both (16–75)	Trial 1: 65.4% Trial 2: 51.8%,	N1, N2	Diagnosis of narcolepsy based on PSG and MSLT performed ˂5 years; Currently experience EDS, cataplexy, and recurrent sleep attacks almost daily for at least 3 months. Women of child-bearing potential were required to use a medically accepted method of birth control unless surgically sterile or 2 years postmenopausal	44 sites internationally	—
	[40]	Retrospective analysis of clinical trial	522	41.7 (13.3,17–68)	—	N1	Diagnosis using ICSD-1, daily lapses into sleep ≥3 months, cataplexy, and mean sleep latency ˂8 min on MSLT	United States	English
	[41]	Retrospective analysis of clinical trial	93	38.7 (12.1, 18–70)	65%	N1, N2	ICSD-2 and ≥10 score on the ESS and a mean baseline MWT sleep latency score of ≤10 min	United States	English
	[42]	Sleep disorders clinic	23	32.0 (10.1, 18–57)	83%	N1, N2	ICSD-1	Mexico	English
	[43]	Sleep disorders clinic	10	15.6 (4.5, —)	20%	N1	ICSD-2 including EDS, cataplexy, confirmation using PSG, and MSLT ≤8 min, with two or more SOREM	Taiwan	Chinese
ESS-CHAD	[19]	Sleep clinics	29	11.6 (3.5, 7–17)	48%	N1	Diagnosed with N1, with ICSD criteria cited	United States	English
	[32]	Retrospective analysis of clinical trial	106	11.9 (2.39, 7–16)	40%	N1	ICSD-2 or 3, depending on when participant was diagnosed or undergoes an MSLT to confirm type 1 using ICSD-3 criteria. Exclusion: various (e.g. unstable medical condition, inability to follow instructions)	United States (inc. several internationally)	English
NSS	[25]	Sleep clinic/university	175	41.5 (17.4)	41%	N1	ICSD-3, cataplexy, mean sleep latency on MSLT ≤8 min with ≤2 sleep-onset REM periods and CSF hypocretin-1 level <110 pg/mL	France	French
	[29]	Sleep clinic	122	26.1 (15.4)	34%	—	ICSD-3 criteria (N1)	China	Chinese
	[44]	Sleep clinic/university	381	38.9 (17.1, —)	47%	N11	ICSD-3, cataplexy, mean sleep latency on MSLT ≤8 min with ≤2 sleep-onset REM periods and CSF hypocretin-1 level <110 pg/mL	France	French
	[30]	Outpatient clinic	52	37.6 (12.0, 18–70)	60%	N1	ICSD-3	Brazil	Spanish
	[31]	Sleep clinic	151	31.4 (11.5, —)	28%	N1	Diagnosis using ICSD-3, complaints of sleepiness for atleast 3 months, mean sleep latency of MSLT <8 min with ≥2 SOREMPs, hypocretin-1 deficiency (<110 pg/mL, *n* ¼ 37) or, if CSF hypocretin-1 unavailable, clear-cut cataplexy, and positive HLADQB1*0602	China	Chinese
NSS-P	[26]	Sleep clinic	209	13.3 (2.6, 6–17)	41%	N1	Diagnosis using ICSD-3, presence of EDS for at least 3 months, mean sleep latency ≤8 min MSLT with at least 2 sleep-onset REM periods, and typical cataplexy, or low CSF levels of orexin-A (<110 pg/mL).	France	French
PDSS-C	[27]	Sleep disorders clinic	31	12.6 (3.4, —)	32%	N1	Diagnosis using the ICSD-2, diagnosis of narcolepsy with cataplexy using clinical interviews (confirmed by MSLT and PSG scores and human leukocyte antigen [HLA] typing of DQB1*0602 positive)	China	Chinese
PSQI-K	[45]	Regional sleep disorder clinic	50	26.7 (12.7, —)	44%	N1, N2	ICSD-2	Korea	Korean
SSS	[46]	Sleep disorder clinic	10	42 (—, 19–65)	70%	N1	Sleep attacks and cataplexy	—	—

### Evaluating the evidence base supporting the use of PROMs in a narcolepsy population using the COSMIN methodology

A pooled summary of the findings from psychometric studies included in this analysis can be found in Supplementary C, Table S4.

1) Content validity

We found only one study that explored content validity; an evaluation of the Epworth Sleepiness Scale—Children and Adolescence (ESS-CHAD) [19]. Another briefly described the development process of the Narcolepsy Severity Scale (NSS) [25]. No other PROMs, including the widely used ESS, had a published study evaluating the content validity in an adult narcolepsy population. Table 3 summarizes the appraisal of content validity using the COSMIN guidelines.

**Table 3.**
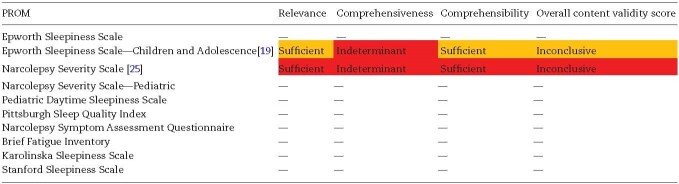
Content validity of PROMs used in RCTs of people with narcolepsy

Content validity results obtained in these studies were rated against COSMIN criteria for what is considered evidence of good content validity (sufficient, insufficient, inconclusive). The background color of each cell represents our confidence that the results obtained in these studies reflect the true content validity of the PROM, as assessed using the COSMIN GRADE approach (green = high, yellow = moderate, orange = low, red = very low). — A dash indicates no evidence was found assessing this measurement property.

#### ESS-CHAD

No development study was found for the ESS-CHAD (or for the ESS upon which it was based). The content validity study of the ESS-CHAD explored the relevance and comprehensibility of the items but not comprehensiveness [19]. Relevance, comprehensiveness, and comprehensibility are equally important, and all three are required; thus, the ESS-CHAD received an overall content validity rating of “inconclusive.” Quality of evidence was found to be low due to the small size of the study population (*n* = 13 children, *n* = 19 adolescents), concerns that changes made to the ESS-CHAD following this study were not assessed, and the number of researchers involved in analyzing the qualitative interviews not described.

#### NSS

No content validity studies were found for the NSS; however, one publication briefly described the development process [25]. While the paper briefly discussed the relevance and comprehensibility of the items, comprehensiveness was not mentioned. Overall, the quality of evidence was rated very low (due to the brief description), and overall content validity was rated inconclusive.

2) Structural validity

COSMIN defines structural validity as a measure of the degree to which the scores of a PROM are an adequate reflection of the dimensionality of the construct being measured. If a PROM has sufficient structural validity, the whole PROM should be unidimensional (i.e. all items measure a single construct), or the PROM should contain subscales (where all items in a subscale measure a unidimensional construct).

**Figure 4.**
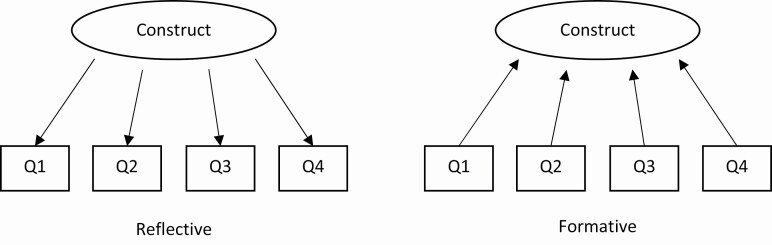
Conceptual diagram representing the relationship between questions and the construct measured in reflective and formative question models.

The requirement for sufficient structural validity only applies to PROMs that are based on a reflective questionnaire model. In a reflective model, all questions are manifestations of the same construct (i.e. the questions reflect aspects of a single construct) (Figure 4). Conversely, a formative model is where the construct does not exist naturally on its own and is instead “formed” from different constructs (Figure 4). The General Anxiety Disorder-7 (GAD-7) is an example of a reflective model, as all questions measure manifestations of anxiety (a single construct). Conversely, the Pittsburgh Sleep Quality Index (PSQI) is an example of a formative model, as it contains subscales measuring different aspects of sleep (e.g. sleep duration, sleep disturbances) that are combined into a single construct of sleep quality. Structural validity is an important measurement property for reflective models as we expect questions measuring a single construct to be related, whereas it has no meaning in a formative model as there is no requirement for questions or constructs measured to be related to one another [10, 14, 28].

#### NSS and NSS-P

The structural validity of the NSS and NSS-P was explored in several studies [25, 29–31]. However, these PROMs are designed as a single scale that purports to measure a construct of symptom severity that does not naturally exist (i.e. they are “formative” models that assess the five different symptoms of narcolepsy (e.g. severity of EDS, severity of cataplexy) and combined into a single dimension).

3) Internal consistency

Internal consistency refers to the interrelatedness of items within a unidimensional scale or subscale, measured using Cronbach’s α. For internal consistency to be correctly understood and interpreted, sufficient evidence of structural validity is required as a prerequisite (i.e. scale is unidimensional or has subscales) [14]. Subscale internal consistency can be shown for PROMs based on formative models if the PROM subscale is unidimensional and all items within a subscale measure the one construct) [14].

#### ESS-CHAD

Internal consistency of the ESS-CHAD was assessed in a single study using retrospective clinical trial data [32]. Using an N1 population (*n* = 100), Cronbach’s α was 0.76 (95% CI: 0.68–0.82). This score was rated indeterminant for internal consistency as no evidence of structural validity of the ESS-CHAD (or ESS) in a narcolepsy population was found (considered a prerequisite for proper interpretation of the score) [14]. While structural validity has been explored in other cohorts, other reviews did find consensus on this psychometric property of the ESS [33].

#### NSS and NSS-P

Internal consistency of the NSS and NSS-P was evaluated in the same papers as the construct validity [25, 29–31] and assessed either between all questions or between questions grouped by the results of factor analysis. As neither the NSS and NSS-P measure a unidimensional construct nor contain subscales, the measurement property of internal consistency was considered irrelevant (Table 4 and Supplementary Table S4). As per the COSMIN checklist, the findings were summarized but not scrutinized [14].

**Table 4.**
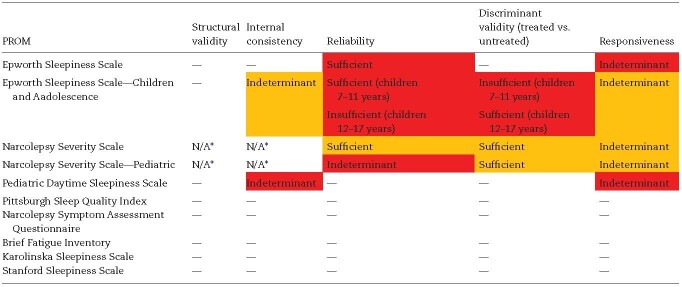
Summary of other measurement properties of PROMs used in narcolepsy RCTs

Pooled results from each measurement property of each PROM were rated against COSMIN criteria for what is considered evidence of good measurement property (sufficient, insufficient, inconclusive). The background color of each cell represents our confidence that the results obtained in these studies reflect the true content validity of the PROM, as assessed using the COSMIN GRADE approach (green = high, yellow = moderate, orange = low, red = very low). — A dash indicates no evidence was found assessing this measurement property.

*An N/A rating was given where a measurement property was assessed in a study, but the measurement property was found to not be relevant. As per the COSMIN checklist, structural validity and internal consistency are irrelevant to PROMs that are based on formative question models.

#### PDSS

Internal consistency of the PDSS was explored in one study using a narcolepsy population [27]. While internal consistency was found to be sufficient (Cronbach’s α = 0.81), it lacked evidence of structural validity in a narcolepsy population and thus rated indeterminant (Table 4 and Supplementary Table S4). The quality of evidence was graded very low due to the small population size (*n* = 31).

4) Test–retest reliability

Test–retest reliability refers to the proportion of total variance in a respondent’s PROM scores that is due to “true” differences between patients. It is a measure of the consistency of the score rather than its accuracy, and its proper interpretation of the statistic relies on the assumption that the respondent’s symptoms are stable across time points [34].

#### ESS

The test–retest reliability of the ESS was measured in two studies that retrospectively analyzed RCT studies [35, 36]. Scores were compared across different time points in the RCT, with the population size of each analysis varying (lowest *n* = 52, highest *n* = 199). A pooled result of ICC: 0.81–0.87 was reported and rated sufficient against the criteria for good measurement properties (Table 4 and Supplementary Table S4). The quality of the evidence was graded “very low” due to the RCT setting, as proper interpretation requires patients to be stable across time points (stability was assumed, no evidence reported), concerning RCT participants not being representative of the narcolepsy population (due to clinical trial inclusion/exclusion criteria with one study requiring ESS score of >14) and potential incorporation bias [37].

#### ESS-CHAD

Test–retest reliability of the ESS-CHAD was explored using retrospective analysis of clinical trial data [32]. Children and adolescents (*n* = 64) were assessed, with an ICC: 0.76 reported. When separated by age, sufficient test–retest reliability was reported in children of 7–11 years (*n* = 21) (ICC: 0.86), yet found to be insufficient in children of 12–17 years (*n* = 43) (ICC: 0.66). Like the ESS, evidence was rated very low due to the RCT setting, clinical trial population not necessarily representative of the wider population, and small population size.

#### NSS

Test–retest reliability of the NSS was explored across four studies using a narcolepsy population [25, 29–31]. A total population of 86 persons diagnosed with N1 participated, and the pooled ICC: 0.71–0.92 was rated as sufficient against the criteria for good measurement properties (Table 4 and Supplementary Table S4). The quality of evidence was overall graded low due to the small population size and the long time interval between measurements (up to several months where there may have been a considerable change).

#### NSS-P

Test–retest reliability of the NSS-P was explored in a single study of 32 participants diagnosed with N1 [26]. The result showed no significant difference between time points; however, this was rated indeterminant as a dependent *t*-test was used for statistical analysis between time points rather than interclass coefficient or weighted kappa (Table 4 and Supplementary Table S4) [14]. The quality of evidence was graded very low due to the small population size (*n* = 32) and unknown time interval used in the study.

5) Hypothesis for testing construct validity—discriminant validity

Discriminant validity or known-group validity is a measure of the ability of a PROM to distinguish between groups, where the measurement of a specific construct is *a priori* assumed to differ between them (i.e. participants treated for sleepiness should be less sleepy than those who are untreated) [14]. This type of validity relies on the assumption that the PROM validly measures a specific construct.

#### ESS-CHAD

The capacity of the ESS-CHAD to discriminate between treated/non-treated cohorts and between sex in children was assessed through retrospective analysis of clinical trial data (*n* = 100) [32]. A two-tailed *t*-test was used to calculate the mean difference between female/male (−0.68) and nontreated/treated (2.84) participants. Furthermore, analysis showed that in participants aged 7–11 years (*n* = 36), mean difference was assessed between female/male (−1.59) and non-treated/treated (1.30). Similarly, participants aged 12–16 years (*n* = 64), mean difference was also reported between female/male (−0.27) and non-treated/treated (3.39). We found the ESS insufficient for discriminative validity in children under 12 and sufficient for those aged 12–17 years. Quality of evidence was rated very low due to the population used (clinical trial participants who may not be representative of the entire population), incorporation bias, and small cohort size.

#### NSS

Discriminant validity of the NSS was explored in three studies using 637 people with N1 [25, 29, 44]. A *t*-test was used to determine the mean difference between treated/non-treated adults (mean difference: 9.08, 7.70, and 4.60). The NSS was able to distinguish between medicated and non-medicated individuals (*p* < 0.05), however quality of evidence was graded low due to the mix of interventions used and the structure of the PROM weighted towards the symptom EDS (i.e. we are unable to tell if the PROM can discriminate between people treated/untreated for single symptom domains like cataplexy) (Supplementary Table 4, S4).

#### NSS-P

Discriminant validity of the NSS-P was explored in a single study of 160 participants diagnosed with N1 [26]. The NSS-P was able to distinguish between non-treated/treated individuals (mean difference = 3.71). (*p* < 0.05), with quality of evidence was graded low due to similar concerns raised in NSS (Supplementary Table 4, S4).

6) Responsiveness to change (in response to intervention)

Responsiveness is the ability of a PROM to detect a change in a construct before and after an intervention. The result for this measurement property is rated using hypothesis testing, where authors determined a priori the size and direction of the effect a treatment would have on a PROM score [14]. This is typically informed by a minimal clinically important difference (MCID), the minimum threshold for an outcome score that a patient or physician would consider a given change to be meaningful or worthwhile [47]. This is typically calculated using anchor points (other reference points or outcomes such as QoL measures) that show that an intervention has clinical significance. An MCID for any PROM is needed to adequately assess its responsiveness psychometric. It is common for psychometric studies to use a paired *t*-test to show the responsiveness, however, this is considered inappropriate. A paired *t*-test shows that a statistically significant difference exists between the mean scores of a PROM pre- and post-intervention (i.e. H_0_ = PROM score pre- and post-intervention is the same). Showing significance using a paired *t*-test does not assess if the magnitude of the difference in scores is clinically significant (informed by the MCID) [14, 48].

#### ESS

Responsiveness of the ESS was explored in a single study consisting of 10 adults and children diagnosed with N1 [43]. The study found the ESS was able to show a statistically significant difference in means pre- and post-treatment; however, this was rated indeterminant due to the use of a paired *t*-test and no evidence of an MCID used in the study. Quality of evidence was rated as very low due to the small population size and participants being a mix of adults/children, which is considered inappropriate due to differences in the presentation of narcolepsy in these two groups [49–51].

#### ESS-CHAD

A retrospective analysis of clinical trial data was used to explore the responsiveness of the ESS-CHAD in children (<18 years) diagnosed with N1 (*n* = 59) [32]. The study found the ESS-CHAD was able to show a statistically significant difference in means pre- and post-treatment; however, this was rated indeterminant due to the use of a paired *t*-test and no evidence of an MCID used in the study. This contributed to a quality of evidence rating of very low, along with the small population size (7–10 cohort, *n* = 21).

#### NSS

Four studies explored the responsiveness of the NSS using 160 participants diagnosed with N1 [25, 30, 31, 44]. Pooled results showed a statistically significant difference of means between pre- and post-treatment scores using the NSS; however, this was calculated using paired *t*-test. Confidence intervals for the difference of means nor ΔSD were provided in any of these studies. No MCID for the NSS was found; thus, responsiveness was rated indeterminant. The quality of evidence was rated low due to a mix of interventions given to participants and the small population size of each study. This is because the NSS does not contain subscales and is weighted more towards measuring EDS symptoms (75% of questions relate to EDS). It is unknown if the NSS is responsive to change when measuring interventions targeting symptoms other than EDS.

#### NSS-P

A single study explored the responsiveness of the NSS-P using 33 participants diagnosed with N1 [26]. Pooled results showed sufficient responsiveness of the NSS, with a mean difference in score of 3.12 ± 7.12 reported between treated/ untreated cohorts. The study did suggest an MCID of 3.60–3.76; however, this was calculated using effect sizes (e.g. 0.5 × ΔSD), not in combination with any anchor points. This is not considered an appropriate calculation of MCID and thus is not a reflection of what people with narcolepsy would consider clinically significant [52]. This, along with the use of a paired *t*-test, informed our rating of indeterminate. The quality of evidence was rated low due to the small population size and the mix of interventions given to participants, similarly seen in studies of responsiveness of the NSS (Supplementary Table 4, S4).

#### PDSS

A single study explored the responsiveness of the PDSS using 31 participants diagnosed with N1 [27]. The study indicated that the PDSS could detect change over time, but no results were published, thus rated as indeterminant. The quality of evidence was rated as very low due to the small population size and lack of information published in the study (Supplementary Table 4, S4)

7) Hypothesis for testing construct validity—convergent validity

Convergent validity refers to how closely the PROM relates to other variables and measured constructs. In the context of narcolepsy, convergent validity can be difficult to interpret as some constructs are not well defined (i.e. ESS and MSLT measuring different aspects of sleepiness). Thus convergent validity was not assessed using the COSMIN (as per checklist), instead summarized qualitatively (Supplementary Table S4).

#### ESS

The ESS measures a different construct than its objective counterparts (i.e. MSLT and MWT) (Supplementary Table S5) [53]. We hypothesized *a priori* that there should be a strong negative correlation with the MWT and a strong positive correlation with the MSLT (considering all used as measures of sleepiness in EDS). Pooling the results of validation studies together, we found the correlation was smaller than expected (MWT *r* = −0.42 to −0.18, MSLT *r* = 0.41 to 0.27).

#### NSS

The NSS was compared against the ESS, MWT, MSLT, and PSQI. While these outcome measures capture different constructs, a moderate, positive correlation with the ESS was expected and reflects that approximately 50% of the NSS questions relate to sleepiness/vigilance.

8) Cultural validity, measurement error, and measurement invariance

No validation studies exploring cultural validity/measurement invariance and measurement error in a narcolepsy population were found. Criterion validity was not included in this study as no there is no gold standard of narcolepsy that PROMs could be compared against.

## Discussion

The first stage of this systematic review identified the ESS (a PROM) as the most frequent outcome measure used in narcolepsy RCTs, followed in frequency by objective measures: the MWT and PSG. When assessing outcome measures used in narcolepsy child/adolescent RCTs, only four RCTs were found to have used a specific pediatric population. The clinical global impressions (change) were used four times, while cataplexy diaries, the MSLT, and the PDSS were all used twice. The modified version of the ESS designed for children and adolescents (ESS-CHAD) was used once as a secondary measure.

Overall, we identified ten PROMs used in either two or more RCTs or developed specifically to measure symptom/disability in people with narcolepsy. In the second stage of this review, we found very little evidence supporting the use of these 10 PROMs in RCTs measuring treatment efficacy in people with narcolepsy. Most PROMs assessed excessive daytime sleepiness (EDS), with few assessing other symptoms associated with narcolepsy [4]. Few high-quality psychometric studies were found assessing these PROMs, with concerns around sample size, incorporation bias, and inappropriate statistical tests identified.

### Content validity and the construct EDS

Content validity is considered the most important psychometric property as it refers to how well a PROM measures all aspects of a given construct. Our analysis showed that PROMs used to capture excessive daytime sleepiness in narcolepsy trials lacked evidence of content validity. This may be because of the way they construct of EDS is conceived. The definition of EDS varies across the literature (including academic and regulatory approval documentation), with “EDS” and “excessive sleepiness” often used interchangeably. A recent review describes EDS presenting clinically as several sleep-related symptoms (e.g. excessive sleepiness, sleep attacks, sleep inertia, etc.), while people with narcolepsy have stressed their experience of EDS extends beyond just sleepiness to include autonomic functions and cognition [53]. If EDS is a multidimensional construct, clarity is needed around how best to capture these dimensions. Our review found that both objective and subjective outcome measures purporting to assess EDS as the primary endpoint in RCTs (i.e. MWT, ESS, and MSLT) assessed dimensions of actual sleepiness. Perhaps other dimensions of EDS should be used as the outcome in RCTs to better reflect patient concerns, as treatment may only be efficacious for excessive sleepiness but not sleep attacks or potentially less efficacious for this aspect of EDS than others. Variability in the items assessing EDS makes it difficult to compare treatment efficacies, as frequently used PROMs and objective measures in RCTs capture different aspects of sleepiness.

There was little variation in outcome measures used to capture cataplexy, with weekly cataplexy diaries commonly used. However, these diaries preclude the assessment of many measurement properties due to the lack of standardization of items and responses and fail to capture nuances of the symptom (i.e. partial/full cataplexy attacks, whether residual cataplexy is tolerable) [4].

No specific outcome measures were identified for the other symptoms of narcolepsy.

### Patient-reported outcome measures

#### ESS and ESS-CHAD

The ESS was the most frequently used outcome measure in RCTs in people with narcolepsy and the second-most frequently used primary outcome measure. Despite its frequency of use and acceptance by regulatory authorities, we found surprisingly little evidence supporting its use in people with narcolepsy. No content validity studies were found for the ESS in adults, nor were studies found exploring structural validity and internal consistency using an adult narcolepsy population. There was evidence (from low-quality studies) for the convergent validity between the ESS and MSLT/MWT, which demonstrated a weaker-than-expected correlation, yet all three outcome measures have been used as the primary endpoint for EDS in narcolepsy RCTs. Validity is the degree to which a PROM measures the construct it purports to measure, and given the frequency of use of the ESS in clinical trials (*n* = 49), it’s remarkable that limited quality studies have been completed. Only one study showed sufficient evidence of responsiveness to change; however, this was graded “very low” quality as the study population used was small (*n* = 10) and comprised of a mix of adults and children (considered inappropriate as an adult and pediatric narcolepsy differ in clinical presentation and severity) [43, 50, 51].

Most studies on measurement properties of the ESS in people with narcolepsy were retrospective analyses of RCTs. This includes two studies that showed sufficient test–retest reliability of the ESS; however, the quality of this evidence was rated very low. Inclusion/exclusion criteria of clinical trials are selective, and this needs to be taken into consideration when appraising validation studies that use this data. The cohort used should be representative of all those with narcolepsy, not an ideal clinical trial population (e.g. inclusion criteria of one RCTs used in a validation study required an ESS score of ≥14, mean sleep latency of MWT <10 min, and women required to be on birth control, while also excluding many comorbidities [18]). Incorporation bias is also introduced when using RCTs for such studies, whereby the outcome measures are also used as the screening criteria, which may falsely lead to elevated sensitivity [37].

#### ESS-CHAD

The ESS-CHAD was one of two PROMs used in child/adolescent narcolepsy RCTs. Content validity was explored in one study, with sufficient relevance and comprehensibility shown, but comprehensiveness was not explored. Assessment of discriminatory validity in children 7–11 years found a mean difference of 1.30 between untreated/treated cohorts, whereas, in children 12–17 years, the mean difference was 3.39 between untreated/treated. It is unclear if a score of 1.30 is a MCID, with the result perhaps attributed to the advanced reading skill needed to interpret the items of the PROM; we calculated that a seventh-grade reading level is required (Flesch Reading Ease Score: 73.5) [54]. It may be that most children under 12 do not understand the difference between a “high chance of falling asleep” and a “moderate chance of falling asleep.” Sufficient test–retest reliability was shown in children under 12 (ICC: 0.856), with insufficient test–retest reliability in children 12–17 years (ICC: 0.656). Given concerns around the interpretability of the ESS-CHAD, it is reasonable to assume older children would have a higher test–retest score than the younger cohort; however, this was not observed. This may be attributed to the small population size used in under 12 years (*n* = 8 untreated/*n* = 13 treated) and calls for further validation studies to be undertaken.

#### NSS (adult and pediatric)

Conversely, we found several validation studies of the NSS. Development was briefly detailed in Dauvilliers et al. and validated for use in an N1 population [25, 26], but no content validity studies were found for either the NSS or the NSS-P. There are some concerns around the comprehension of the NSS-P, as one study stated that responses from 20% of participants were excluded from the study as they misunderstood the question/symptoms [26]. The NSS was created to assess the traditional “five symptoms” of narcolepsy, with a final combined score reflective of overall symptom severity. Yet the NSS/NSS-P does not contain subscales, thus limiting its ability to evaluate change in the different symptoms of narcolepsy and limiting its applicability to N1 when it could also be used in an N2 population. Allowing the five symptom domains to be scored as individual scales would allow the assessment of individual symptoms whilst allowing for subscales to be assessed for appropriate measurement properties and combined into an overall final score. Further validation studies could be conducted using this format and across the five symptom domains (e.g. assessing responsiveness to EDS treatment, responsiveness to cataplexy treatment).

#### Other PROMs

The evidence base for psychometric properties of other PROMs used in narcolepsy trials was either very limited or completely lacking.

#### Summary

Based on the results of this review, no PROM can be recommended as a measure of treatment efficacy in a narcolepsy population. The ESS and ESS-CHAD purport to measure “average sleep propensity.” However, evidence suggests they may not be appropriate for use as an endpoint for EDS, as patients have reported EDS extends beyond sleepiness [4, 53]. High-quality psychometric property studies that are not retrospective analyses of clinical trials are needed to inform several psychometric aspects, particularly construct validity. To inform the property of responsiveness, identification of a MCID using anchor points (e.g. patient and/or clinician-based determinants of “change” or improvement) are required, as has been done with other conditions (e.g. depression) [52]. Conversely, the NSS and NSS-P contain questions related to EDS that extend beyond sleepiness, with “daytime sleep attacks” and “worry” around falling asleep throughout the day assessed. Neither the NSS nor NSS-P can be recommended for assessing treatment efficacy in RCTs as the PROM results in a final score comprised of five narcolepsy symptom domains combined. This raises questions about its appropriateness for assessing an intervention that only targets one symptom. The addition of subscales for each symptom and further psychometric testing are recommended.

### Research agenda/future prospective

To accurately assess treatment efficacy in narcolepsy, EDS and other symptoms first need to be clearly defined in narcolepsy phenotypes (i.e. N1/N2, adult/child). Persons with narcolepsy have indicated in several forums that EDS extends beyond the feeling of sleepiness [4, 53]. Furthermore, work is needed to clarify these dimensions through qualitative study and extends to other symptom domains such as cataplexy. Only then can appropriate outcome measures be chosen or developed to accurately capture change in these domains.

To ensure PROMs used in narcolepsy RCTs are appropriate for use, both quality psychometric studies of existing PROMs and perhaps the development of PROMs specific to narcolepsy are needed. This includes validated measures for assessing cataplexy as diaries may not be able to distinguish from similar phenomena (e.g. cataplexy mimicries such as epilepsy) [4]. Given the context of treatment efficacy in RCTs, priority should be given to the development of MCIDs using anchor points that are meaningful to people with narcolepsy (e.g. HR-QoL, ability to work, etc). This would allow for a better understanding of the responsiveness of each PROM in use.

## Conclusion

This systematic review identified the most common outcome measures used in RCTs in narcolepsy populations and assessed the psychometric properties of PROMs used. While the ESS is the most common outcome measure used in RCTs of narcolepsy treatments, there seems to be remarkably little evidence of its psychometric properties. Given the primacy of the ESS, a thorough validation study of its measurement properties seems overdue. Further study is needed around what aspects of EDS and other symptoms are important to people with narcolepsy before we determine how best to measure these. Our study points to the need for comprehensive PROMs to be developed for narcolepsy (tailored for subtypes and adults/children), as well as further high-quality validation studies of existing PROMs. Furthermore, identification of a minimal clinically important difference is needed from the patient perspective for each PROM before we can be confident that we are accurately measuring the symptoms experienced by persons with narcolepsy and to what extent interventions are efficacious.

## Supplementary Material

zsac156_suppl_Supplementary_Material
