# Supplementary material for: Evaluation of psychometric properties of patient-reported outcome measures frequently used in narcolepsy randomized controlled trials: a systematic review
Source: Sleep. 2022 Jul 7;45(10):zsac156. doi: 10.1093/sleep/zsac156 (PMC9548672; doi:10.1093/sleep/zsac156)
Supplement: zsac156_suppl_Supplementary_Material [file zsac156_suppl_supplementary_material.docx]

**Title:** Evaluation of psychometric properties of patient-reported outcome measures frequently used in narcolepsy randomized controlled trials: a systematic review

**Authors:**

Aaron Schokman ^1,2^

Yu Sun Bin ^2^

Diana Naehrig ^1^

Janet M.Y Cheung ^3^

Kristina Kairaitis ^4,5^

Nick Glozier ^1^

**Affiliations**

^1^ Central Clinical School, The Faculty of Medicine and Health, The University of Sydney, NSW, 2006, Australia

^2^ Sleep Theme, Charles Perkins Centre, University of Sydney, Camperdown, NSW, Australia, yusun.bin@sydney.edu.au

^3^ School of Pharmacy, Faculty of Medicine and Health, The University of Sydney, NSW, 2006, Australia

^4^ Department of Respiratory and Sleep Medicine, The University of Sydney at Westmead Hospital, Westmead, NSW, Australia kristina.kairaitis@sydney.edu.au

^5^ Ludwig Engel Centre for Respiratory Research, Westmead Institute for Medical Research, Westmead, NSW

Corresponding author: Aaron Schokman, Office 8, Level 5, Professor Marie Bashir Centre, Missenden Road, Camperdown, NSW, 2050, Australia, aaron.schokman@sydney.edu.au

**Supplementary A**

**Search Strategy S1:**

**Online clinical trial records**

**ClinicalTrial.gov (combined records from search 1 and 2)**

- Search 1
  - Condition/Disease: Narcolepsy
  - Study Type: Interventional Studies (Clinical Trials)
- Search 2
  - Condition/Disease: Cataplexy
  - Study Type: Interventional Studies (Clinical Trials)

**Clinicaltrialsregister.eu**

- Disease: Narcolepsy

**anzctr.org.au**

- Narcolepsy

**Bibliographic Databases**

**Medline**

1. (narcole* or cataple* or gelineau* or anti cataple* or anticataple*).tw. or exp Narcolepsy/ or exp Cataplexy/
2. (randomized controlled trial or controlled clinical trial).pt. or randomized.ab. or placebo.ab. or drug therapy.fs. or randomly.ab. or trial.ab. or groups.ab.
3. exp animals/ not humans.sh.
4. 2 not 3
5. 1 and 4

**Embase**

1. (narcole* or cataple* or gelineau* or anti cataple* or anticataple*).tw. or exp Narcolepsy/ or exp Cataplexy/
2. randomized controlled trial/ OR Controlled clinical study/ OR random$.ti,ab. OR randomization/ OR intermethod comparison/ OR placebo.ti,ab. OR (compare or compared or comparison).ti. OR ((evaluated or evaluate or evaluating or assessed or assess) and (compare or compared or comparing or comparison)).ab. OR (open adj label).ti,ab. OR ((double or single or doubly or singly) adj (blind or blinded or blindly)).ti,ab. OR double blind procedure/ OR parallel group$1.ti,ab. OR (crossover or cross over).ti,ab. OR ((assign$ or match or matched or allocation) adj5 (alternate or group$1 or intervention$1 or patient$1 or subject$1 or participant$1)).ti,ab. OR (assigned or allocated).ti,ab. OR (controlled adj7 (study or design or trial)).ti,ab. OR (volunteer or volunteers).ti,ab. OR OR human experiment/ OR trial.ti.
3. (random$ adj sampl$ adj7 (cross section$ or questionnaire$1 or survey$ or database$1)).ti,ab. not (comparative study/ or controlled study/ or randomi*ed controlled.ti,ab. or randomly assigned.ti,ab.) OR Cross-sectional study/ not (randomized controlled trial/ or controlled clinical study/ or controlled study/ or randomi?ed controlled.ti,ab. or control group$1.ti,ab.) OR (((case adj control$) and random$) not randomi*ed controlled).ti,ab. OR (Systematic review not (trial or study)).ti.OR (nonrandom$ not random$).ti,ab. OR Random field$.ti,ab. OR (random cluster adj3 sampl$).ti,ab. OR (review.ab. and review.pt.) not trial.ti. OR (we searched.ab. and review.ti.) or review.pt.OR update review.ab. OR (databases adj4 searched).ab. OR ((rat or rats or mouse or mice or swine or porcine or murine or sheep or lambs or pigs or piglets or rabbit or rabbits or cat or cats or dog or dogs or cattle or bovine or monkey or monkeys or trout or marmoset$1).ti. and animal experiment/) OR Animal experiment/ not (human experiment/ or human/)
4. 2 not 3
5. 1 and 4

**PSYCHINFO**

1. "treatment effectiveness evaluation".tw. OR exp treatment outcomes/ OR placebo.tw. OR followup studies.tw. OR placebo*.tw. OR random*.tw. OR comparative stud*.tw. OR (clinical adj3 trial*).tw. OR (research adj3 design).tw. OR (evaluat* adj3 stud*).tw. OR (prospectiv* adj3 stud*).tw. OR ((singl* or doubl* or trebl* or tripl*) adj3 (blind* or mask*)).tw.
2. (narcole* or cataple* or gelineau* or anti cataple* or anticataple*).tw. or exp Narcolepsy/ or exp Cataplexy/
3. 1 and 2
4. limit 3 to (journal article and human)

**CINAHL**

(MH randomized controlled trials OR MH double-blind studies OR MH single-blind studies OR MH random assignment OR MH pretest-posttest design OR MH cluster sample OR TI (randomised OR randomized) OR AB (random*) OR TI (trial) OR (MH (sample size) AND AB (assigned OR allocated OR control)) OR MH (placebos) OR PT (randomized controlled trial) OR AB (control W5 group) OR MH (crossover design) OR MH (comparative studies) OR AB (cluster W3 RCT)) NOT ((MH animals+ OR MH animal studies OR TI animal model*) NOT MH human) AND (TX narcole* OR TX cataple* OR TX gelineau* OR TX anticataple*)

**SCOPUS**

( TITLE-ABS-KEY ( narcolep* OR cataple* OR gelineau* OR anti‐cataple* OR anticataple* ) ) AND ( TITLE-ABS-KEY ( ( clinic* W/1 trial* ) OR ( randomi* W/1 control* ) OR ( randomi* W/2 trial* ) OR ( random* W/1 assign* ) OR ( random* W/1 allocat* ) OR ( control* W/1 clinic* ) OR ( control* W/1 trial ) OR placebo* OR ( quantitat* W/1 stud* ) OR ( control* W/1 stud* ) OR ( randomi* W/1 stud* ) OR ( singl* W/1 blind* ) OR ( singl* W/1 mask* ) OR ( doubl* W/1 blind* ) OR ( doubl* W/1 mask* ) OR ( tripl* W/1 blind* ) OR ( tripl* W/1 mask* ) OR ( trebl* W/1 blind* ) OR ( trebl* W/1 mask* ) ) AND NOT ( SRCTYPE ( b ) OR SRCTYPE ( k ) OR SRCTYPE ( p ) OR SRCTYPE ( r ) OR SRCTYPE ( d ) OR DOCTYPE ( ab ) OR DOCTYPE ( bk ) OR DOCTYPE ( ch ) OR DOCTYPE ( bz ) OR DOCTYPE ( cr ) OR DOCTYPE ( ed ) OR DOCTYPE ( er ) OR DOCTYPE ( le ) OR DOCTYPE ( no ) OR DOCTYPE ( pr ) OR DOCTYPE ( rp ) OR DOCTYPE ( re ) OR DOCTYPE ( sh ) ) )

**Search Strategy S2:**

**MEDLINE**

1. (instrumentation or methods).fs. OR (Validation Studies or Comparative Study).pt. OR exp Psychometrics/ OR psychometr*.ti,ab. OR (clinimetr* or clinometr*).tw. OR exp Outcome Assessment Health Care/ OR outcome assessment.ti,ab. OR outcome measure*.tw. OR exp Observer Variation/ OR observer variation.ti,ab. OR exp Health Status Indicators/ OR exp Reproducibility of Results/ OR reproducib*.ti,ab. OR exp Discriminant Analysis/ OR (reliab* or unreliab* or valid* or coefficient or homogeneity or homogeneous or internal consistency).ti,ab. OR (cronbach* and (alpha or alphas)).ti,ab. OR (item and (correlation* or selection* or reduction*)).ti,ab. OR (agreement or precision or imprecision or precise values or test-retest).ti,ab. OR (test and retest).ti,ab. OR (reliab* and (test or retest)).ti,ab. OR (stability or interrater or inter-rater or intrarater or intra-rater or intertester or inter-tester or intratester or intra-tester or interobserver or inter-observer or intraobserver or intraobserver or intertechnician or inter-technician or intratechnician or intra-technician or interexaminer or inter-examiner or intraexaminer or intra-examiner or interassay or interassay or intraassay or intra-assay or interindividual or inter-individual or intraindividual or intra-individual or interparticipant or inter-participant or intraparticipant or intra-participant or kappa or kappas or kappas or repeatab*).ti,ab. OR ((replicab* or repeated) and (measure or measures or findings or result or results or test or tests)).ti,ab. OR (generaliza* or generalisa* or concordance).ti,ab. OR (intraclass and correlation*).ti,ab. OR (discriminative or known group or factor analysis or factor analyses or dimension* or subscale*).ti,ab. OR (multitrait and scaling and (analysis or analyses)).ti,ab. OR (item discriminant or interscale correlation* or error or errors or individual variability).ti,ab. OR (variability and (analysis or values)).ti,ab. OR (uncertainty and (measurement or measuring)).ti,ab. OR (standard error of measurement or sensitiv* or responsive*).ti,ab. OR ((minimal or minimally or clinical or clinically) and (important or significant or detectable) and (change or difference)).ti,ab. OR (small* and (real or detectable) and (change or difference)).ti,ab. OR (meaningful change or ceiling effect or floor effect or Item response model or IRT or Rasch or Differential item functioning or DIF or computer adaptive testing or item bank or cross-cultural equivalence).ti,ab.
2. (narcole* or cataple* or gelineau* or anti cataple* or anticataple*).tw. or exp Narcolepsy/ or exp Cataplexy/
3. see note
4. 1 and 2 and 3

Note: This line was substituted with search parameters of each patient reported outcome measure: (epworth sleepiness scale.mp,tw,ab.), (narcolepsy severity scale.mp,tw,ab.), (stanford sleepiness scale.mp,tw,ab.), (karolinska sleepiness scale.mp,tw,ab.), (pittsburgh sleep quality index.mp,tw,ab.), (paediatric daytime sleepiness scale.mp,tw,ab.), (brief fatigue inventory.mp,tw,ab.), (narcolepsy symptom assessment questionnaire.mp,tw,ab.)

**Embase**

1. exp intermethod comparison/ or exp data collection method/ or exp validation study/ or exp feasibility study/ or exp pilot study/ or exp psychometry/ or exp reproducibility/

2. (reproducib* or audit or psychometr* or clinimetr* or clinometr*).ab,ti. or exp observer variation/ or observer variation.ab,ti. or exp discriminant analysis/ or exp validity/ or reliab*.ab,ti. or valid*.ab,ti. or coefficient.ab,ti. or internal consistency.ab,ti. or (cronbach* and (alpha or alphas)).ab,ti. or item correlation.ab,ti. or item correlations.ab,ti. or item selection.ab,ti. or item selections.ab,ti. or item reduction.ab,ti. or item reductions.ab,ti. or agreement.ab,ti. or precision.ab,ti. or imprecision.ab,ti. or precise values.ab,ti. or test-retest.ab,ti. or (test and retest).ab,ti. or (reliab* and (test or retest)).ab,ti. or stability.ab,ti. or interrater.ab,ti. or inter-rater.ab,ti. or intrarater.ab,ti. or intra-rater.ab,ti. or intertester.ab,ti. or inter-tester.ab,ti. or intratester.ab,ti. or intratester.ab,ti. or interobeserver.ab,ti. or inter-observer.ab,ti. or intraobserver.ab,ti. or intraobserver.ab,ti. or intertechnician.ab,ti. or inter-technician.ab,ti. or intratechnician.ab,ti. or intratechnician.ab,ti. or interexaminer.ab,ti. or inter-examiner.ab,ti. or intraexaminer.ab,ti. or intraexaminer.ab,ti. or interassay.ab,ti. or inter-assay.ab,ti. or intraassay.ab,ti. or intra-assay.ab,ti. or interindividual.ab,ti. or inter-individual.ab,ti. or intraindividual.ab,ti. or intra-individual.ab,ti. or interparticipant.ab,ti. or inter-participant.ab,ti. or intraparticipant.ab,ti. or intraparticipant.ab,ti. or kappa.ab,ti. or kappas.ab,ti. or coefficient of variation.ab,ti. or repeatab*.ab,ti. or ((replicab* or repeated) and (measure or measures or findings or result or results or test or tests)).ab,ti. or generaliza*.ab,ti. or generalisa*.ab,ti. or concordance.ab,ti. or (intraclass and correlation*).ab,ti. or discriminative.ab,ti. or known group.ab,ti. or factor analysis.ab,ti. or factor analyses.ab,ti. or factor structure.ab,ti. or factor structures.ab,ti. or dimensionality.ab,ti. or subscale*.ab,ti. or multitrait scaling analysis.ab,ti. or multitrait scaling analyses.ab,ti. or item discriminant.ab,ti. or interscale correlation.ab,ti. or interscale correlations.ab,ti. or ((error or errors) and (measure* or correlat* or evaluat* or accuracy or accurate or precision or mean)).ab,ti. or individual variability.ab,ti. or interval variability.ab,ti. or rate variability.ab,ti. or variability analysis.ab,ti. or (uncertainty and (measurement or measuring)).ab,ti. or standard error of measurement.ab,ti. or sensitiv*.ab,ti. or responsive*.ab,ti. or (limit and detection).ab,ti. or minimal detectable concentration.ab,ti. or interpretab*.ab,ti. or (small* and (real or detectable) and (change or difference)).ab,ti. or meaningful change.ab,ti. or minimal important change.ab,ti. or minimal important difference.ab,ti. or minimally important change.ab,ti. or minimally important difference.ab,ti. or minimal detectable change.ab,ti. or minimal detectable difference.ab,ti. or minimally detectable change.ab,ti. or minimally detectable difference.ab,ti. or minimal real change.ab,ti. or minimal real difference.ab,ti. or minimally real change.ab,ti. or minimally real difference.ab,ti. or ceiling effect.ab,ti. or floor effect.ab,ti. or item response model.ab,ti. or irt.ab,ti. or rasch.ab,ti. or differential item functioning.ab,ti. or dif.ab,ti. or computer adaptive testing.ab,ti. or item bank.ab,ti. or cross-cultural equivalence.ab,ti.

3. (narcole* or cataple* or gelineau* or anti cataple* or anticataple*).tw. or exp Narcolepsy/ or exp Cataplexy/

4. see note

5. 1 and 2 and 3 and 4

Note: This line was substituted with search parameters of each patient reported outcome measure: (epworth sleepiness scale.mp,tw,ab.), (narcolepsy severity scale.mp,tw,ab.), (stanford sleepiness scale.mp,tw,ab.), (karolinska sleepiness scale.mp,tw,ab.), (pittsburgh sleep quality index.mp,tw,ab.), (paediatric daytime sleepiness scale.mp,tw,ab.), (brief fatigue inventory.mp,tw,ab.), (narcolepsy symptom assessment questionnaire.mp,tw,ab.)

**PSYCHINFO**

1. (randomized controlled trial or controlled clinical trial).pt. or randomized.mp,tw,ab. or placebo.mp,tw,ab. or drug therapy.sh. or randomly.mp,tw,ab. or trial.mp,tw,ab. or groups.mp,tw,ab.

2. (narcole* or cataple* or gelineau* or anti cataple* or anticataple*).tw. or exp Narcolepsy/ or exp Cataplexy/

3. see note

4. 1 and 2 and 3

5. limit 4 to (journal article and human)

Note: This line was substituted with search parameters of each patient reported outcome measure: (epworth sleepiness scale.mp,tw,ab.), (narcolepsy severity scale.mp,tw,ab.), (stanford sleepiness scale.mp,tw,ab.), (karolinska sleepiness scale.mp,tw,ab.), (pittsburgh sleep quality index.mp,tw,ab.), (paediatric daytime sleepiness scale.mp,tw,ab.), (brief fatigue inventory.mp,tw,ab.), (narcolepsy symptom assessment questionnaire.mp,tw,ab.)

**Scopus:**

( TITLE-ABS-KEY ( see note ) AND TITLE-ABS-KEY ( narcolep* OR cataple* OR gelineau* OR anti‐cataple* OR anticataple* ) )

Note: This search parameter was substituted of each patient reported outcome measure: (epworth AND sleepiness AND scale), (narcolepsy AND severity AND scale), (stanford AND sleepiness AND scale), (karolinska AND sleepiness AND scale), (pittsburgh AND sleep AND quality AND index), (paediatric AND daytime AND sleepiness AND scale), (brief AND fatigue AND inventory), (narcolepsy AND symptom AND assessment AND questionnaire)

**Supplementary B**

Tables S1, S2 and S3 are replications under a Creative Commons Attribution 4.0 International License (<http://creativecommons.org/licenses/by/4.0/>) of Prinsen CAC, Mokkink LB, Bouter LM, et al. COSMIN guideline for systematic reviews of patient-reported outcome measures. Qual Life Res. 2018;27(5):1147-1157. doi:10.1007/s11136-018-1798-3.

| Measurement property | Raiting^1^ | **Criteria** |
| --- | --- | --- |
| Structural validity | + | **CTT:**  CFA: CFI or TLI or comparable measure >0.95 OR RMSEA  <0.06 OR SRMR <0.082  **IRT/Rasch**:  No violation of unidimensionality3: CFI or TLI or comparable  measure >0.95 OR RMSEA <0.06 OR SRMR <0.08  *AND*  no violation of local independence: residual correlations  among the items after controlling for the dominant factor <  0.20 OR Q3's < 0.37  *AND*  no violation of monotonicity: adequate looking graphs OR item  scalability >0.30  *AND*  adequate model fit:  IRT: χ2 >0.01  Rasch: infit and outfit mean squares ≥ 0.5 and ≤ 1.5 OR Z-standardized values > ‐2 and <2 |
|  | ? | CTT: Not all information for ‘+’ reported  IRT/Rasch: Model fit not reported |
|  | - | Criteria for ‘+’ not met |
| Internal consistency | + | At least low evidence4 for sufficient structural validity5 AND  Cronbach's alpha(s) ≥ 0.70 for each unidimensional scale or  subscale6 |
|  | ? | Criteria for “At least low evidence4 for sufficient structural  validity5” not met |
|  | - | At least low evidence4 for sufficient structural validity5 AND  Cronbach’s alpha(s) < 0.70 for each unidimensional scale or  subscale6 |
| Reliability | + | ICC or weighted Kappa ≥ 0.70 |
|  | ? | ICC or weighted Kappa not reported |
|  | - | ICC or weighted Kappa < 0.70 |
| Measurement error | + | SDC or LoA < MIC5 |
|  | ? | MIC not defined |
|  | - | SDC or LoA > MIC5 |
| Hypotheses testing for  construct validity | + | The result is in accordance with the hypothesis7 |
|  | ? | No hypothesis defined (by the review team) |
|  | - | The result is not in accordance with the hypothesis7 |
| Cross‐cultural  validity\measurement  invariance | + | No important differences found between group factors (such  as age, gender, language) in multiple group factor analysis OR  no important DIF for group factors (McFadden's R2 < 0.02) |
|  | ? | No multiple group factor analysis OR DIF analysis performed |
|  | - | Important differences between group factors OR DIF was  found |
| Criterion validity | + | Correlation with gold standard ≥ 0.70 OR AUC ≥ 0.70 |
|  | ? | Not all information for ‘+’ reported |
|  | - | Correlation with gold standard < 0.70 OR AUC < 0.70 |
| Responsiveness | + | The result is in accordance with the hypothesis7 OR AUC ≥ 0.70 |
|  | ? | No hypothesis defined (by the review team) |
|  | - | The result is not in accordance with the hypothesis7 OR AUC <  0.70 |

Table S1: AUC = area under the curve, CFA = confirmatory factor analysis, CFI = comparative fit index, CTT= classical test theory, DIF = differential item functioning, ICC = intraclass correlation coefficient, IRT = item response theory, LoA = limits of agreement, MIC = minimal important change, RMSEA: Root Mean Square Error of Approximation, SEM = Standard Error of Measurement, SDC = smallest detectable change, SRMR: Standardized Root Mean Residuals, TLI = Tucker‐Lewis index

1 “+” = sufficient, ” –“ = insufficient, “?” = indeterminate, 2 To rate the quality of the summary score, the factor structures should be equal across studies, 3 Unidimensionality refers to a factor analysis per subscale, while structural validity refers to a factor analysis of a (multidimensional) patient‐reported outcome measure, 4 As defined by grading the evidence according to the GRADE approach, 5 This evidence may come from different studies, 6 The criteria ‘Cronbach alpha < 0.95’ was deleted, as this is relevant in the development phase of a PROM and not when evaluating an existing PROM. 7 The results of all studies should be taken together

Table S2: Modified Grade Approach for grading quality of evidence: Scores are rated in seriousness based on COSMIN standards.

| Quality of evidence | Lower evidence score if |
| --- | --- |
| High | Risk of bias  -1 Serious  -2 Very serious  -3 Extremely serious  Inconsistency  -1 Serious  -2 Very serious  Imprecision  -1 total n = 50-100  -2 total n < 50  Indirectness  -1 Serious  -2 Very serious |
| Moderate |  |
| Low |  |
| Very Low |  |

Table S3: COSMIN definition of quality levels used to rate the quality of evidence.

| Quality level | Definition |
| --- | --- |
| High | We are very confident that the true measurement property lies close to that of the estimate* of the measurement property |
| Moderate | We are moderately confident in the measurement property estimate: the true measurement property is likely to be close to the estimate of the measurement property, but there is a possibility that it is substantially different |
| Low | Our confidence in the measurement property estimate is  limited: the true measurement property may be substantially  different from the estimate of the measurement property |
| Very low | We have very little confidence in the measurement property  estimate: the true measurement property is likely to be  substantially different from the estimate of the measurement  property |

* Estimate of the measurement property refers to the pooled or summarized result of the measurement property of a PROM.

**SUPPLEMENTARY C**

Table S4: Summary of published evidence of frequently used PROMs in narcolepsy RCTs, assessed using the COSMIN guidelines

| **Psychometric property** | **Summary (pooled) of findings from psychometric studies** | **Quality of methodology used in studies** | **Confidence that findings in studies accurately reflect the true psychometric property** |
| --- | --- | --- | --- |
| **Structural validity** |  |  |  |
| NSS | Four studies with a combined population of 500. Three studies used principal components factor analysis (3 factors explaining 54.86% variance, 3 factors explaining 58% variance and 5 factors explaining 75.2% variance) and one used exploratory factor analysis (3 factors explaining 54.86% variance. N/A rating as PROM is based on formative model. ^1-4^ | N/A | N/A |
| NSS-P | A single study of 160 participants. Principal component factor analysis found 4 factors explaining 61.95% of variance. N/A rating as PROM is based on formative model. ^5^ | N/A | N/A |
| **Internal Consistency** |  |  |  |
| ESS-CHAD | Single study of 100 children/adolescents <18 years of age. Cronbach’s α: 0.76 (95% CI: 0.68 – 0.82). Indeterminant rating as no evidence of structural validity found in narcolepsy population (limited evidence of structural validity of ESS in other populations). ^6^ | Indeterminant | Low |
| NSS | Five studies with combined population of 719, internal consistency between questions and overall score: Cronbach’s α = 0.75 – 0.86. N/A rating as PROM is based on formative model. ^1-4^. | N/A | N/A |
| NSS-P | Single study of 160 participants: Cronbach’s α = 0.74 – 0.84. N/A rating as PROM is based on formative model ^5^. | N/A | N/A |
| PDSS | A single study of 31 participants, Cronbach’s α = 0.81. Indeterminant rating as no evidence of structural validity found in narcolepsy population.^7^ | Indeterminant | Very low |
| **Reliability** |  |  |  |
| ESS | Two studies where reliability was assessed at different timepoints in several cohorts (cohort size varied between 52 and 199). A pooled ICC: 0.81 – 0.87 was reported. Both studies were retrospective analyses of clinical trial data, with one study having ESS score of >14 as inclusion criteria. ^8,9^ | Sufficient | Very Low |
| ESS-CHAD | Single study of 64 participants under 18 years of age with result of ICC: 0.76 (95% CI: 0.63 – 0.84). Separated by age, children aged 7-11 (n = 32) had ICC: 0.86 (95% CI: 0.68 – 0.94) and adolescents aged 12 – 17 years had ICC: 0.66 (95% CI: 0.45 – 0.80). This study was a retrospective analysis of clinical trial data. ^6^ | Sufficient (children aged 7 - 11) | Very Low |
|  |  | Insufficient (adolescents aged 12 -17) |  |
| NSS | Four studies with a combined population of 86. Each study had a small population (n < 50). A pooled ICC: 0.71 – 0.92 was reported ^1-4,10^. | Sufficient | Low |
| NSS-P | A single study of 32 participants, a dependent t-test showed no significant difference between two-time points however this was rated indeterminant as ICC or weighted kappa not reported ^5^ | Indeterminant | Very Low |
| **Discriminant Validity** |  |  |  |
| ESS-CHAD | Single study of 100 children/adolescents under 18 years of age compared scores of an untreated and treated cohort. The mean difference in scores was 2.84 (95% CI: 1.14, 4.55). Separated by age , the 7 – 11 cohort (n = 36) had a mean difference of 1.30 (95% CI: -2.53, 5.13). In cohort aged 12 – 17 (n = 64), mean difference was 3.39 (95% CI: 1.54, 5.24). Study was retrospective analysis of clinical trial data. ^6^ | Insufficient (children aged 7 - 11) | Very Low |
|  |  | Sufficient (adolescents aged 12 -17) |  |
| NSS | Three studies with a combined population of 637. NSS was able to distinguish between treated/untreated cohorts however as multiple interventions were used, and the PROM assesses 5 symptom domains, we were unable to determine if it could discriminate between treated/untreated when treating specific symptoms. Thus evidence was rated low ^1,3,10^ | Sufficient | Low |
| NSS-P | A single study consisting of 160 participants. The NSS-P was able to distinguish between treated/untreated cohorts (significant mean difference between groups) however as multiple interventions were used, and the PROM assesses 5 symptom domains, we were unable to determine if it could discriminate between treated/untreated when treating specific symptoms. Thus evidence was rated low ^5^ | Sufficient | Low |
| **Responsiveness** |  |  |  |
| ESS | Single study (n = 10) consisting of a mix of adults and children. Mean difference in score between treated/untreated was 6.20. No minimal clinically important difference was found; thus responsiveness could not be properly assessed.^11^ | Indeterminant | Very Low |
| ESS-CHAD | Single study of 59 children/adolescents under 18 years of age compared scores of the same cohort pre and post treatment. The mean difference in score was 6.31. Separated by age, the 7 – 11 cohort (n = 21) had a mean difference in score was 5.67. In cohort aged 12 – 17 (n = 38), mean difference in score was 6.66. No minimal clinically important difference was found. Study was a retrospective analysis of clinical trial data.^6^ | Indeterminant | Low |
| NSS | Four studies with a combined population of 160 participants. Mean difference in score pre- and post- treatment was 5.70 – 8.27 (pooled). No minimal clinically important difference was found; thus, responsiveness could not be properly assessed.^1,2,4,10^ | Indeterminant | Low |
| NSS-P | A single study of 33 participants. Mean difference in score pre- and post- treatment was 5.70 – 8.27 No minimal clinically important difference was found; thus responsiveness could not be properly assessed. ^5^ | Indeterminant | Low |
| PDSS | A single study consisting of 31 participants. The study indicated PDSS could detect change over time but statistical evidence not published. ^7^ | Indeterminant | Very low |

ESS: Epworth Sleepiness Scale, E-CHAD: Epworth Sleepiness Scale-Children and Adolescent, NSS: Narcolepsy Severity Scale, NSS-P: Narcolepsy Severity Scale-Paediatric, PDSS: Paediatric Daytime Sleepiness Scale (PDSS)

Table S5: Summary of evidence of convergent validity of objective and subjective measures of EDS

|  | **Summary (pooled) of findings from psychometric studies** | **Quality of methodology used in studies**  **(Sufficient / Indeterminant / Insufficient)** | **Confidence that findings from studies accurately reflect the true psychometric property** |
| --- | --- | --- | --- |
| **ESS** |  |  |  |
| MSLT | Three studies showed weak, negative correlation (r = -0.41 – -0.27) ^12-14^ | **Sufficient** | **Moderate** |
| MWT | Three studies showed weak, negative correlation (r = -0.42 – -0.18) ^13,15,16^ | **Sufficient** | **Low** |
| **NSS** |  |  |  |
| ESS | Four studies (n=226) showed moderate-high, positive correlation between measures (r = 0.45 – 0.65) ^1-4^ | **Sufficient** | **Moderate** |
| MWT | One study (n = 24) showed weak, negative correlation (r = -0.30) ^1^ | **Sufficient** | **Low** |
| MSLT | Two studies (n = 88) showed weak, negative correlation (r = -0.41 – -0.21) ^1,2^ | **Sufficient** | **Low** |

ESS: Epworth sleepiness scale, MSLT: Multiple Sleep Latency Test, MWT: Maintenance of Wakefulness Test, NSS: Narcolepsy Severity Scale

1. Dauvilliers Y, Beziat S, Pesenti C, et al. Measurement of narcolepsy symptoms: The Narcolepsy Severity Scale. *Neurology.* 2017;88(14):1358-1365.

2. Li C, Spruyt K, Zhang C, et al. Reliability and validity of the Chinese version of Narcolepsy Severity Scale in adult patients with narcolepsy type 1. *Sleep medicine.* 2021;81:86-92.

3. Ouyang H, Han F, Zheng Q, Zhang J. Chinese version of narcolepsy severity scale: a validation study. *BMC neurology.* 2019;19(1):334.

4. Pimentel Filho LH, Gomes ACD, Frange C, Tufik S, Coelho FMS. Validation of the Brazilian Portuguese version of the narcolepsy severity scale. *Sleep medicine.* 2020;76:134-139.

5. Barateau L, Lecendreux M, Chenini S, et al. Measurement of Narcolepsy Symptoms in School-Aged Children and Adolescents: The Pediatric Narcolepsy Severity Scale. *Neurology.* 2021;97(5):e476-e488.

6. Wang YG, Menno D, Chen A, et al. Validation of the Epworth sleepiness scale for children and adolescents (ESS-CHAD) questionnaire in pediatric patients with narcolepsy with cataplexy aged 7–16 years. *Sleep Medicine.* 2022;89:78-84.

7. Yang CM, Huang YS, Song YC. Clinical utility of the Chinese version of the Pediatric Daytime Sleepiness Scale in children with obstructive sleep apnea syndrome and narcolepsy. *Psychiatry and clinical neurosciences.* 2010;64(2):134-140.

8. van der Heide A, van Schie MKM, Lammers GJ, et al. Comparing Treatment Effect Measurements in Narcolepsy: The Sustained Attention to Response Task, Epworth Sleepiness Scale and Maintenance of Wakefulness Test. *Sleep.* 2015;38(7):1051-1058.

9. Rosenberg R, Babson K, Menno D, et al. Test-retest reliability of the Epworth Sleepiness Scale in clinical trial settings. *Journal of sleep research.* 2021:e13476.

10. Dauvilliers Y, Barateau L, Lopez R, et al. Narcolepsy Severity Scale: a reliable tool assessing symptom severity and consequences. *Sleep.* 2020;43(6).

11. Yeh S-B, Schenck CH. Efficacy of modafinil in 10 Taiwanese patients with narcolepsy: findings using the Multiple Sleep Latency Test and Epworth Sleepiness Scale. *The Kaohsiung journal of medical sciences.* 2010;26(8):422-427.

12. Jiménez-Correa U, Haro R, Obdulia González R, Velázquez-Moctezuma J. Correlations between subjective and objective features of nocturnal sleep and excessive diurnal sleepiness in patients with narcolepsy. *Arquivos de neuro-psiquiatria.* 2009;67:995-1000.

13. Sangal RB, Mitler MM, Sangal JM. Subjective sleepiness ratings (Epworth sleepiness scale) do not reflect the same parameter of sleepiness as objective sleepiness (maintenance of wakefulness test) in patients with narcolepsy. *Clinical neurophysiology.* 1999;110(12):2131-2135.

14. Komada Y, Inoue Y, Mukai J, Shirakawa S, Takahashi K, Honda Y. Difference in the characteristics of subjective and objective sleepiness between narcolepsy and essential hypersomnia. *Psychiatry and clinical neurosciences.* 2005;59(2):194-199.

15. Weaver TE, Mathias SD, Crosby RD, et al. Relationship between sleep efficacy endpoints and measures of functional status and health‐related quality of life in participants with narcolepsy or obstructive sleep apnea treated for excessive daytime sleepiness. *Journal of sleep research.* 2021;30(3):e13210.

16. Erman M, Emsellem H, Black J, Mori F, Mayer G. Correlation between the Epworth Sleepiness Scale and the Maintenance of Wakefulness Test in patients with narcolepsy participating in two clinical trials of sodium oxybate. *Sleep medicine.* 2017;38:92-95.
